# Clinical withdrawal symptom profile of synthetic cannabinoid receptor agonists and comparison of effects with high potency cannabis

**DOI:** 10.1007/s00213-021-05945-1

**Published:** 2021-09-17

**Authors:** Sam Craft, Jason A. Ferris, Monica J. Barratt, Larissa J. Maier, Michael T. Lynskey, Adam R. Winstock, Tom P. Freeman

**Affiliations:** 1 Addiction and Mental Health Group (AIM), Department of Psychology, University of Bath, Bath, UK; 2 National Addiction Centre, Institute of Psychiatry, Psychology and Neuroscience, King’s College London, London, UK; 3 Centre for Health Services Research, University of Queensland, QLD, Queensland Brisbane, Australia; 4 Social and Global Studies Centre and Digital Ethnography Research Centre, RMIT University, Victoria Melbourne, Australia; 5 National Drug and Alcohol Research Centre, University of New South Wales Sydney, New South Wales Sydney, Australia; 6 Department of Clinical Pharmacy, University of California San Francisco, San Francisco, USA; 7 Global Drug Survey Ltd, London, UK; 8 Institute of Epidemiology and Health Care, University College London, London, UK

**Keywords:** Synthetic cannabinoids, Spice, SCRA, Withdrawal, Cannabis, Effect profile, Abuse liability

## Abstract

Synthetic cannabinoid receptor agonists (SCRAs) may be used as an alternative to natural cannabis; however, they may carry a greater risk of problematic use and withdrawal. This study aimed to characterise the withdrawal symptom profile of SCRAs and compare their profile of effect with high-potency herbal cannabis. Global Drug Survey data (2015 and 2016) were used to access a clinically relevant sample of people reporting use of SCRAs >10 times in the past 12-months, a previous SCRA quit attempt, and lifetime use of high-potency herbal cannabis. Participants completed an 11-item SCRA withdrawal symptom checklist and compared SCRAs and high-potency herbal cannabis on their onset and duration of effects, speed of the development of tolerance, severity of withdrawal, and difficulty with dose titration. Participants (*n* = 284) reported experiencing a mean of 4.4 (95% CI: 4.1, 4.8) withdrawal symptoms after not using SCRAs for >1 day; most frequently reported were sleep issues (59.2%), irritability (55.6%), and low mood (54.2%). Withdrawal symptoms were significantly associated with frequency (>51 vs. 11–50 times per year: IRR = 1.43, 95% CI: 1.16, 1.77, *p* = 0.005) and quantity (grams per session: IRR = 1.13, 95% CI: 1.05, 1.22, *p* = 0.001) of SCRA use. Compared to high-potency herbal cannabis, SCRAs were rated as having a faster onset and shorter duration of effects, faster development of tolerance, and more severe withdrawal (*p*’s < 0.001). In conclusion, SCRA withdrawal symptoms are more likely to occur after greater SCRA exposure. The effects of SCRA indicate a more severe withdrawal syndrome and a greater risk of problematic use than natural cannabis.

## Introduction

Synthetic cannabinoid receptor agonists (SCRAs) were first identified in branded herbal smoking mixtures in 2008 (Auwärter et al. 2009). The first identified compound, JHW-018, was rapidly followed by other SCRA compounds and they now represent the largest group of novel psychoactive substances (NPS) monitored by the EU Early Warning System with over 200 different SCRAs currently being monitored (EMCDDA 2019b). Although functionally similar to delta-9-tetrahydrocannabinol (THC)—the primary psychoactive component of cannabis—showing activity at both cannabinoid type 1 receptors (CB1Rs) and cannabinoid type 2 receptors (CB2Rs), structurally, SCRAs are a diverse group of chemicals varying widely in potency, receptor affinity, and effect profile (EMCDDA 2017). Also, while THC acts as a partial agonist, SCRAs are typically full receptor agonists, often binding to CB1Rs with greater affinity and efficacy, producing pharmacological effects between 2 and 100 times more potent than THC (Castaneto et al. 2014).

Whilst there are licenced cannabinoid-based medicinal products which are produced synthetically (e.g. Dronabinol and Nabilone), these are not typically classified as SCRAs, which, by contrast, are used exclusively for recreational purposes and do not fall under descriptions of medicinal products (e.g. under common EU Law; UNODC 2015). Mostly manufactured by groups of clandestine chemists or chemical companies based in China, SCRAs are typically synthesised as powders, dissolved in solvent (e.g. acetone), and sprayed onto inert herbal material (EMCDDA 2017). Sold under brand names such as “Spice” or “K2”, the appearance of these products closely resemble natural herbal cannabis and they are often consumed in a similar way (e.g. smoked in joints with or without tobacco or in a bong; Castaneto et al. 2014; Gunderson et al. 2014). Though less common, SCRAs may also be sold as resin or as pure powders, and more recently oils, e-liquids, and impregnated paper/card which can be used in vapes or e-cigarettes (Angerer et al. 2019; EMCDDA 2017; Ford and Berg 2018; Norman et al. 2020). Not only is there significant variation in composition between these products, but due to crude and unstandardised production processes, there can also be considerable variability within batches. During production, drug material may be unevenly distributed across the base material, resulting in highly concentrated “hot pockets” or “hotspots” of SCRA compounds. Dosing of SCRAs is therefore highly inconsistent, conferring a greater risk of overdose and other adverse effects (EMCDDA 2017).

As a result of their high potency, variation in product composition, and resultant difficulty in titrating drug effect, SCRAs have been identified as carrying a much greater risk of acute harm than natural cannabis (Winstock et al. 2015). Despite attempts to prohibit their sale through various legislative acts, SCRAs have continued to cause problems in many countries including New Zealand (Macfarlane and Christie 2015), the USA (Monte et al. 2017), and the UK (particularly in custodial settings; Ralphs et al. 2017). To date, most research has focused on acute harms and the toxicity associated with their consumption, where extreme agitation and aggression, cardiovascular/respiratory risks, neurological excitation (including seizures), acute psychotic symptoms, and other adverse psychological outcomes are seen (Akram et al. 2019; Cooper 2016; Hermanns-Clausen et al. 2013; Tait et al. 2016; Waugh et al. 2016). Adverse effects are reported even at relatively low levels of use, and although severe effects are seen among those with greater levels of SCRA exposure (e.g. Durand et al. 2015; Schep et al. 2015; Ustundag et al. 2015), there is currently limited research on how amount or frequency of use influence outcomes (Akram et al. 2019).

Also, despite increasing evidence of acute harm, less is known about the longer-term effects of chronic SCRA use, including dependence or withdrawal. Cannabis use disorder is currently one of the most prevalent substance use disorders worldwide (Degenhardt et al. 2018) and higher potency products confer greater risk (Craft et al. 2020; Hines et al. 2020; Freeman and Winstock 2015). Also, although cannabis withdrawal syndrome was only included in the most recent edition of the Diagnostic and Statistical Manual of Mental Disorders, DSM-5 (American Psychiatric Association 2013)—there is consistent evidence that cannabis withdrawal symptoms are highly prevalent among people who frequently use cannabis and strongly correlated with cannabis use disorder (Budney et al. 1999; Livne et al. 2019; for a review see Bonnet and Preuss 2017). Therefore, higher rates of dependence and a greater risk of withdrawal would be expected with frequent use of more potent receptor agonists; however, these aspects of SCRA are not well described in the literature.

Previously, SCRAs have been reported as having a more rapid onset and shorter duration of effects relative to cannabis (Winstock and Barratt 2013b). However, liability to develop problematic use may be influenced by other drug effects, including the rate at which tolerance is developed, difficulty with dose titration, and severity of withdrawal. Also, despite accumulating case reports detailing various rapidly developing adverse effects associated with withdrawal from SCRA use in small clinical samples (Grigg et al. 2019; Macfarlane and Christie 2015; Nacca et al. 2013; Van Hout and Hearne 2017), not all people who use SCRAs may seek or need medical treatment, and the extent and profile of withdrawal symptoms have not previously been assessed in a larger non-treatment seeking sample.

The aim of this study was to characterise the withdrawal symptom profile of SCRAs and to examine the influence of quantity and frequency of use on the likelihood of experiencing withdrawal symptoms. A secondary aim was to compare the effects of SCRAs with high-potency herbal cannabis across a range of indicators of liability to problematic use among respondents reporting the use of both substances. High-potency herbal cannabis (i.e. sinsemilla/skunk) is produced from intensely cultivated and indoor-grown female plants which are prevented from fertilisation such that THC concentrations are much higher than traditional outdoor-grown herbal cannabis (~ 17.4% vs. 9.8%; Chandra et al. 2019; Potter et al. 2018). It is the most common cannabis preparation globally (EMCDDA 2019a; Freeman et al. 2021; Smart et al. 2017) and has been associated with an increased risk of cannabis use disorder symptomology and psychotic disorders (Craft et al. 2020; Di Forti et al. 2019; Freeman and Winstock 2015; Hines et al. 2020). There appears to be substantial overlap between those that use SCRAs and natural cannabis products (Barratt et al. 2013; Vandrey et al. 2012), and SCRAs may be used as a cannabis substitute, particularly among those attempting to evade detection (e.g. in urine drug screens; Bonar et al. 2014; Gunderson et al. 2014; Loeffler et al. 2016; Vandrey et al. 2012)). Comparing the profile of effects to high-potency cannabis, of which the clinical significance of outcomes is more well known can therefore help to characterise the risk of harm posed by SCRAs.

## Methods

### Sample

The Global Drug Survey (GDS) uses an online platform to conduct anonymous and encrypted annual international surveys in partnership with global media and harm reduction organisations. In 2015 and 2016, it was translated into 10 languages and was promoted in over 20 countries. The GDS is self-completed and as such represents a convenience sample of people who use legal and/or illegal psychoactive substances. Due to its non-probability sample, analyses are suited to identifying specific target populations as opposed to determining the prevalence of drug use within the general population (Barratt et al. 2017). As such, it is ideally suited to profiling new drugs and their potential harms and spotting emerging drug trends (Lawn et al. 2014; Winstock et al. 2014).

The current sample was drawn from the combined pool of 181,870 respondents completing GDS 2015 and 2016. In addition to the core battery of questions asked in every annual survey, the GDS also explores a variety of more specific research themes with additional specialist modules that vary over survey years. In 2015 and 2016, a specialist module was added to compare the effects of SCRAs and high potency herbal cannabis. Across these two surveys, *n* = 2916 reported using SCRAs in the last year. In order to identify a clinically relevant sample to investigate SCRA withdrawal symptoms and compare the effect profile of SCRAs with high-potency herbal cannabis, the analyses reported here used a subsample of those who (a) reported using SCRAs more than 10 times in the past 12-months, (b) had previously attempted to quit using SCRAs (for at least one day), and (c) had also used natural high-potency herbal cannabis in their lifetime (*n* = 284). Any respondents from the 2016 dataset who indicated they had completed previous versions of the annual GDS were excluded to ensure the final sample included only unique respondents. Participants accessed the survey through a website link that was promoted widely through global media partners and social media networks such as Facebook and Twitter for a period of 8 weeks in November and December each year. Participation was voluntary and no incentives (payments or lotteries) were offered for participation. Ethical approval was received from King’s College London (PNM1415-18 Global Drug Survey) and University of Queensland (No: 2017001452) Research Ethics Committees.

### Measures

Through GDS 2015 and GDS 2016, a harmonised set of identical questions were asked of participants who reported past 12-months use of SCRAs. These included when they last used SCRAs (asked in categories: within the last 12 months, within the last 30 days, within the last 7 days) number days used in the past 12-months (asked in categories: 11–50, 51–100, >100), typical amount (grams) used per session, the most common route of administration (single response from the following options: bong/water pipe, hot knife, vape pen, vaporiser, joint mixed with tobacco, oral, insufflation) and preparations ever used (yes/no: herbal, resin, powder, oil). Participants were also asked a series of questions about their cannabis use. Regarding cannabis use in general (i.e. any cannabis product), participants were asked the number of days of use in the past 12-months (asked in categories: 1, 2–10, 11–50, 51–100, >100), typical amount used per session (grams), and most common route of administration (single response from the following options: pipe, bong/water pipe, hot knife, vaporiser, joint mixed with tobacco, joint without tobacco, blunt, eaten in food). Then participants were asked when they last used high-potency herbal cannabis specifically (asked in categories: within the last 12 months, within the last 30 days, within the last 7 days). To aid identification of high-potency herbal cannabis (and differentiation from other cannabis products), participants were presented with a series of product-specific-labelled photographs. For examples of images of different cannabis products, see Freeman and Lorenzetti (2020) and Wilson et al. (2019).

Participants were then asked about their experience of SCRA withdrawal. Withdrawal was defined as “a range of unpleasant symptoms experienced when trying to stop using SCRAs” and participants were presented with the following list of withdrawal symptoms and were asked to indicate whether or not they had experienced each symptom after not using SCRAs for more than one day (yes/no: difficulty sleeping, restlessness/irritability, weird dreams, palpitations, craving, low mood, sweatiness, nausea, anger/hostility, agitation and shakiness—adapted from the 10-item cannabis withdrawal discomfort scale (Budney et al. 1999), where nervousness, reduced appetite, and headaches were substituted for sweatiness, nausea, agaitation, and shakiness, based on clinical experience and case reports. The scale showed good internal reliability (Cronbach’s alpha = 0.86 in the current dataset). Finally, to compare SCRAs against high-potency herbal cannabis across 5 domains of liability to problematic use participants were asked the following:

- How difficult is it to titrate the effect you get from SCRAs compared to high-potency herbal cannabis? (Response options: more difficult with SCRAs, more difficult with high-potency herbal cannabis, the same, don’t know).
- How quickly do you get the effects you are seeking from SCRAs compared to high-potency herbal cannabis? (Response options: faster with SCRAs, faster with high-potency herbal cannabis, the same, don’t know).
- How long do the effects last from an equivalent dose of SCRAs compared to high-potency herbal cannabis? (Response options: shorter with SCRAs, shorter with high-potency herbal cannabis, the same, don’t know).
- How quickly do you build up tolerance to SCRAs compared to high-potency herbal cannabis? (Response options: faster with SCRAs, faster with high-potency herbal cannabis, the same, don’t know).
- How would you compare the withdrawal when you stop using SCRA compared to high-potency herbal cannabis (Response options: worse with SCRAs, worse with high-potency herbal cannabis, the same, don’t know).

### Statistical analysis

Unless otherwise stated, missing data for all variables were < 5% of the sample and valid percentages are reported. Mean and standard deviation (SD) or 95% confidence intervals (CI) are provided for normally distributed variables and median and interquartile range (IQR) for skewed variables.

In order to compare participants’ experience of SCRAs with high-potency herbal cannabis across measures of liability to problematic use, chi-squared tests were conducted for the direct comparison between the number of “more difficult/faster/shorter with SCRAs” and “more difficult/faster/shorter with high-potency herbal cannabis” responses. Alpha levels were adjusted based on 5 comparisons using the Bonferroni method (*p* < 0.01). Data for responses of “don’t know” or “the same” were combined for presentation in figures but not included in analyses.

Next, associations between frequency and amount of SCRA use and the number of withdrawal symptoms experienced were tested using a negative binomial regression model (to account for any over-dispersion). Frequency of SCRA use in the past 12-months was dummy coded with the lowest category (11–50 times in the past 12-months) used as a reference. Firstly, the two categories of more frequent use (51–100 times and >100 times in the past 12-months) were entered into the model as separate categories. Then, if these categories were not notably different, they were collapsed into, and entered as, a single category (≥ 51 times in the past 12-months) on the grounds of parsimony. Amount used per session was entered as a continuous variable. The model was also adjusted for age and gender (coded as male = 0, female = 1; one participant identified as transgender and was excluded from the regression model due to the small size within this category). Age was entered as quadratic and cubic terms which were retained if they improved model fit, and interactions between gender and the best fitting age term(s) were also fitted and retained if they improved model fit. Missing data on all variables were excluded using listwise deletion. Only the final model is reported.

## Results

### Sample demographics and SCRA and cannabis use characteristics

In the final sample (*n* = 284), 78.1% of respondents identified as male, 21.5% female, and 0.4% transgender. The median age was 22 (IQR 11) and 85.5% were of white ethnicity. Responses were received from participants residing in 30 countries, including Germany (22.2%), Hungary (20.4%), New Zealand (17.6%), USA (7.4%), and UK (7.4%). For all types of SCRA, the median amount used per session was 0.5 grams (IQR 1.4) and 45.4% of participants reported using between 11–50 times in the past 12-months, 27.1% between 51–100 times, and 27.5% >100 times. Regarding the last time they used SCRAs, 21.8% of participants reported use within the last 7 days, 21.5% within the last 30 days, and 56.7% within the last 12 months. The majority of participants reported either a joint with tobacco (61.6%) or a bong (33.8%) as their most common route of administration (all other administration routes were ~  ≤ 2%) and almost all participants reported use of herbal SCRA preparations in their lifetime (96.8%), whilst the use of resin (10.2%), powder (14.1%), and oil (4.9%) were less common. Most respondents’ (39.1%) last use of high-potency herbal cannabis was within 7 days, 21.8% within 30 days, 17.3% within 12 months, and 21.8% over 12 months ago. Regarding any type of cannabis use, 0.4% of the sample reported using only once in the past 12 months, 6.3% between 2–10 times, 10.0% between 11–50 times, 17.7% between 51–100 times, and 65.7% >100 times, and the median amount used per session was 1 (IQR 1.3) gram. Consistent with SCRAs, the majority of participants reported either a joint with tobacco (56.7%) or bong (20.4%) as their most common routes of cannabis administration.

#### SCRA withdrawal profile and associations with frequency and amount of use

**Fig. 1:**
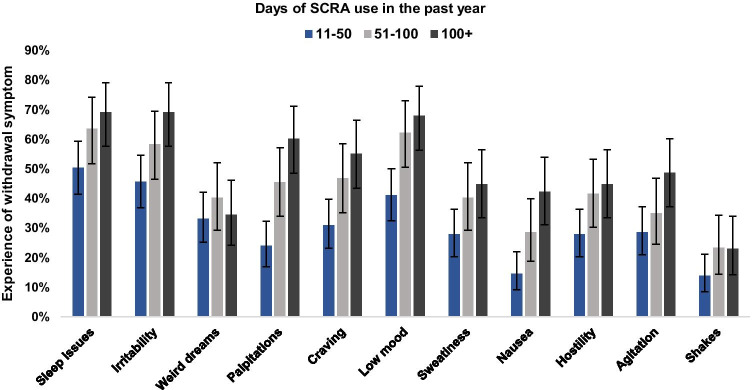
Proportions (95% confidence intervals) of respondents reporting SCRA withdrawal symptoms by frequency of use.

Figure 1 shows the prevalence of SCRA withdrawal symptoms by frequency of use categories. Across the whole sample, the mean number of symptoms reported was 4.4 (95% CI: 4.1, 4.8) with 82.7% reporting at least 1 symptom. The number of participants reporting ≥ 3 and ≥ 4 symptoms were 66.9% and 54.9% respectively, and the most commonly reported symptoms were sleep issues (59.2%), irritability (55.6%), and low mood (54.2%). As summarised in Table 1, the negative binomial regression model (*n* = 260) indicated a significant association between amount and frequency of SCRA use and the number of withdrawal symptoms. In the final and best-fitting model, age was included as a linear term and no age by gender interaction was included. Estimates were not notably different for those using 51–100 and >100 times in the past 12-months, so these two categories were collapsed into ≥51 times on the grounds of parsimony. Compared to those using 11–50 times in the past 12 months, those using ≥51 times experienced a greater number of withdrawal symptoms (incidence rate ratio (IRR): 1.13, 95% CI: 1.05–1.22, *p* = 0.001). Additionally, typical amount of SCRAs used per session in grams was also significantly associated with the number of withdrawal symptoms experienced (IRR: 1.13, 95% CI: 1.05–1.22, *p* = 0.001). After adjustment for the amount of use, age, and gender, these estimates suggest that upon cessation, the probable number of withdrawal symptoms that those using SCRAs 11–50 and ≥51 times in the past 12-months are likely to experience is 3 and 4, respectively.

**Table 1:** Parameter estimates from the final negative binomial regression model showing associations between frequency and quantity of SCRA use and number of withdrawal symptoms experienced in SCRA users who had tried to quit (*n* = 260)

	95% CI			
	IRR	Lower	Upper	*p*
*Total number of withdrawal symptoms* *Pseudo R*^*2*^ = 0.022				
Constant	3.91	2.75	5.56	< 0.001
Frequency of use				
11–50 times	Reference			
≥ 51 times	1.43	1.16	1.77	0.001
Amount used per session (grams)	1.13	1.05	1.22	0.001
Age	0.99	0.98	1.00	0.072
Gender				
Female	Reference			
Male	0.98	0.76	1.25	0.871

Non-linear age terms and age by gender interactions were not retained as they did not improve model fit. As estimates did not suggest a difference, the frequency of use categories 51–100 and >100 times were combined into ≥ 51 times on the grounds of parsimony. *IRR*, incidence rate ratio; 95% CI, 95% confidence intervals for IRR

#### Comparisons between SCRAs and high-potency herbal cannabis

**Fig. 2:**
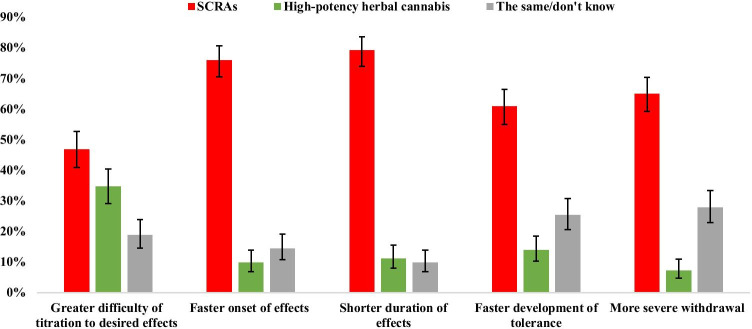
Comparisons between SCRAs and high-potency herbal cannabis across five measures of liability to problematic use (data show proportions and 95% confidence intervals). Note: Between-group comparisons were conducted for SCRA and high potency herbal cannabis responses, the same/don’t know responses were not included in these analyses

Comparisons between participants’ experiences of SCRA and high-potency herbal cannabis across domains of liability to problematic use are shown in Fig. 2. Across each of the measures, SCRAs were rated as having an effect profile which suggests a higher liability to problematic use than natural cannabis. A greater number of people rated SCRAs as having a faster onset of effects (*n* = 238, 88.6% versus 11.3%, *χ*^*2*^_1_ = 142.25, *p* < 0.001), shorter duration of effects (*n* = 252, 87.7% versus 12.3%, *χ*^*2*^_1_ = 143.25, *p* < 0.001), faster development of tolerance (*n* = 210, 81.4% versus 18.6%, *χ*^*2*^_1_ = 82.97, *p* < 0.001), and more severe withdrawal (*n* = 200, 90.0% versus 10.0%, *χ*^*2*^_1_ = 128.00, *p* < 0.001). Despite a trend towards more people rating SCRAs as being more difficult to titrate, this did not reach the Bonferroni corrected threshold (*n* = 210, 57.5% versus 42.5%, *χ*^*2*^_1_ = 4.93, *p* = 0.026).

## Discussion

This study sought to investigate withdrawal symptoms among a non-treatment-seeking sample of people who use SCRAs, and we found that withdrawal symptoms were more likely to occur among those with greater exposure to SCRAs. Specifically, compared to those using SCRAs 11–50 times in the 12-months, those using ≥51 times experienced approximately 1 more withdrawal symptom upon cessation. Additionally, for every additional gram of SCRAs used, the number of withdrawal symptoms increased by around 13%. These associations between frequency and amount of use each accounted for unique variance in the number of withdrawal symptoms experienced, after adjusting for age and gender. This is the first study to examine the contribution of quantity and frequency of SCRA use to withdrawal symptoms, and our findings suggest that people who use SCRAs more frequently and in greater amounts are significantly more likely to experience a greater number of withdrawal symptoms upon cessation, consistent with those reported within clinical settings (Macfarlane and Christie 2015; Nacca et al. 2013).

Furthermore, in a sample of respondents reporting the use of both substances, SCRAs were rated as having increased liability to problematic use compared to high-potency herbal cannabis. Specifically, they were rated as having a faster onset and shorter duration of effects, quicker development of tolerance, and more severe withdrawal. It is also noteworthy that participants reported using less SCRAs per session than natural cannabis products (median (IQR): 0.5 (1.4) grams for SCRAs compared to 1 (1.3) gram for natural cannabis). These findings are consistent with the increased binding affinity and agonist activity of SCRAs at CB1Rs compared to THC (Castaneto et al. 2014) and with previous research comparing the effect profile of SCRAs and high-potency herbal cannabis (Winstock and Barratt, 2013b).

On the basis of our findings, SCRA withdrawal symptoms may be experienced at lower levels of use (with respect to both frequency and amount) than natural cannabis, which supports previous qualitative evidence from a small sample of people using SCRAs (Van Hout and Hearne 2017). Whilst symptom definitions, diagnostic thresholds, and prevalence estimates vary within the natural cannabis withdrawal literature, among adults with frequent use (typically ≥3 days per week/daily or near-daily), previous studies have reported between 12% and 50% experiencing at least 3 symptoms (Hasin et al. 2008; Livne et al. 2019) and 49% (Copersino et al. 2006) and 47% (Castaneto et al. 2014) experiencing at least 4 symptoms, with participants in the latter study also rating those symptoms as severe. In the current study, 66.9% of participants reported at least 3 withdrawal symptoms whilst 54.9% reported at least 4, with symptoms even reported by many of those using less than weekly. Moreover, the profile of SCRA withdrawal described here, while similar to that of cannabis withdrawal—with sleep difficulty, low mood, and craving being predominant (Budney et al. 1999; Livne et al. 2019)—appears to be more physical, with symptoms consistent with elevated sympathetic arousal such as sweating, palpitations, and shakes also reported.

These results therefore add to previous literature highlighting the increased risk of harm associated with SCRAs compared to natural cannabis products (Winstock et al. 2015; Winstock and Barratt 2013a; b). Levels of treatment engagement among people who use SCRAs and other NPS are typically low (Pirona et al. 2017; Ralphs and Gray 2018), and there is likely to be a large unmet treatment need among this group. There are no evidence-based pharmacological detoxification procedures to assist with the management of SCRA use, and data on treatment outcomes among those with SCRA related issues are currently lacking. Future research should seek to address these issues to help improve the clinical management of people using SCRAs. Furthermore, given that non-detection in urine drug screens and accessibility are commonly reported motivations for use (Bonar et al. 2014; Gunderson et al. 2014; Loeffler et al. 2016; Vandrey et al. 2012), it is possible that policies aimed at discouraging cannabis use (e.g. workplace urine drug screens) may be causing greater health harms by inadvertently leading people to use a more harmful drug (e.g. SCRAs) to avoid detection. It is therefore important that prevention messages aimed at cannabis and other drug use also discourage SCRA use and emphasise their potential for greater harm, whilst the effectiveness of workplace urine drug screens and their risk of displacement to non-detectable substances are reviewed.

This study has several limitations and results should be interpreted in light of these. Firstly, the use of a purposive sampling method limits the generalisability of the findings to the general population. However, the utilization of web-based surveys can fill important evidence gaps (Matias et al. 2019), and there are currently no data of this kind from representative studies on SCRA use as such studies are not well suited to recruit a sufficient sample of people who use SCRAs from a general population sample to produce robust and generalizable results. Barratt et al. provide a comprehensive and critical analysis of using non-probability methods adopted by GDS which challenges the widely held view that populations of people who use drugs recruited in such ways fundamentally differ from those recruited through more representative sampling approaches (Barratt et al. 2017). Secondly, assessments relied on self-report so may be subject to recall bias, and comparisons between SCRA and high-potency herbal cannabis on each domain of abuse liability (i.e. tolerance, withdrawal, dose titration, speed/onset of effects) relied on participant self-definition, with no standardised definition provided. Similarly, participants may not have been able to reliably differentiate between their experiences of SCRA and high-potency herbal cannabis on certain domains (e.g. withdrawal), particularly if both substances are commonly co-used. Comparisons were also not able to account for relative differences in the frequency of use of the two substances which may influence individuals’ experience and ratings of the drugs’ effects. However, notably, participants consistently rated SCRA’s effects as more problematic than high-potency herbal cannabis despite past-year frequency of SCRA use being lower than cannabis use in this sample. Therefore, it is likely these effects are understated and may be more pronounced in a sample where SCRA use is more frequent and matched to that of cannabis use. Thirdly, the SCRA withdrawal items included were based on clinical experience with SCRAs and existing literature from natural cannabis withdrawal (Budney et al. 1999) and the items included were rated for their presence and not their severity. However, there is currently no standardised or validated assessment of SCRA withdrawal, and the results here can be used to inform the development of a standardised SCRA withdrawal scale which can account for differences between SCRA and natural cannabis withdrawal, such as a greater physical withdrawal with SCRAs. Fourth, SCRAs represent a diverse and rapidly evolving group of compounds with varying potency, pharmacokinetics, and pharmacodynamics, and we were not able to differentiate between different SCRA compounds in this study. It is possible that different SCRAs carry varying risks of withdrawal and dependence, and future work should look to conduct a more fine-grained analysis of these risks in the range of SCRAs being used. Lastly, in the sample used here, the levels of SCRA use were relatively low. Many of the respondents in this sample reported using SCRAs less than weekly, and the median amount used per session was 0.5 g per session (which is considerably lower than those reported in clinical samples (e.g. Macfarlane and Christie, 2015). We were also unlikely to capture the populations with the riskiest use patterns—such as people in prison or those experiencing homelessness—where different motivations for use, heavier use, higher rates of comorbid mental/physical health problems, and more severe withdrawal profiles are expected. Nonetheless, the high number of SCRA withdrawal symptoms reported in this study should serve as an indicator of the potential severity of these issues among more marginalised populations where SCRA use appears to be increasing, particularly in the UK (Ralphs and Gray 2018; Ralphs et al. 2017).

## Conclusions

In the largest study to investigate SCRA withdrawal symptoms and liability to problematic use, we found that SCRAs may present a greater risk of problematic use than natural cannabis products. Additionally, SCRAs appear to be associated with a clinical withdrawal profile that whilst similar to cannabis, appears to be more severe and has a more marked physical withdrawal profile. Future research should address the current challenges in clinical management of SCRA related issues to help minimise the harm associated with their use. Until then, gradual dose reduction prior to cessation and time limited use of medications that offer symptomatic relief with relatively good short-term safety profiles such as benzodiazepines should be considered.
